# Molecular Characterization of Cancer Associated Fibroblasts in Prostate Cancer

**DOI:** 10.3390/cancers14122943

**Published:** 2022-06-14

**Authors:** Giovanni Vitale, Michele Caraglia, Volker Jung, Jörn Kamradt, Davide Gentilini, Maria Teresa Di Martino, Alessandra Dicitore, Marianna Abate, Pierosandro Tagliaferri, Annalisa Itro, Matteo Ferro, Raffaele Balsamo, Marco De Sio, Gaetano Facchini, Luca Persani, Kai Schmitt, Matthias Saar, Michael Stöckle, Gerhard Unteregger, Silvia Zappavigna

**Affiliations:** 1 Department of Medical Biotechnology and Translational Medicine (BIOMETRA), University of Milan, 20133 Milan, Italy; giovanni.vitale@unimi.it (G.V.); alessandra.dicitore@unimi.it (A.D.); luca.persani@unimi.it (L.P.); 2 Laboratory of Geriatric and Oncologic Neuroendocrinology Research, Istituto Auxologico Italiano (IRCCS), Cusano Milanino, 20095 Milan, Italy; 3 Department of Precision Medicine, University of Campania “L Vanvitelli”, 80138 Naples, Italy; michele.caraglia@unicampania.it (M.C.); marianna.abate@unicampania.it (M.A.); annalisa.itro@unicapania.it (A.I.); 4 Clinic of Urology and Pediatric Urology, University of Saarland, 66421 Homburg, Germany; volker.jung@uks.eu (V.J.); joern.kamradt@hirslanden.ch (J.K.); matthias.saar@uks.eu (M.S.); michael.stoeckle@uks.eu (M.S.); gerhard.unteregger@uks.eu (G.U.); 5 Bioinformatics and Statistical Genomics Unit, Istituto Auxologico Italiano (IRCCS), 20095 Milan, Italy; davide.gentilini@unipv.it; 6 Department of Brain and Behavioral Sciences, University of Pavia, 27100 Pavia, Italy; 7 Department of Experimental and Clinical Medicine, University “Magna Graecia” of Catanzaro, 88100 Catanzaro, Italy; teresadm@unicz.it (M.T.D.M.); tagliaferri@unicz.it (P.T.); 8 Division of Urology, European Institute of Oncology-IRCCS, 20132 Milan, Italy; matteo.ferro@ieo.it; 9 Urology Clinic, Monaldi Hospital, 80125 Naples, Italy; raffaele.balsamo@unicampania.it; 10 Urology Unit, University of Campania “Luigi Vanvitelli”, 80138 Naples, Italy; marco.desio@unicampania.it; 11 UOC of Medical Oncology, ASL NA 2 Nord, “S.M. delle Grazie” Hospital, 80078 Pozzuoli, Italy; ga.facchi@libero.it; 12 Laboratory of Endocrine and Metabolic Research, Istituto Auxologico Italiano (IRCCS), 20095 Milan, Italy; 13 Department of Pathology, Saarland University Medical Center, 66421 Homburg, Germany; kai.schmitt@uks.eu

**Keywords:** androgen signaling, cancer-associated fibroblasts, cell proliferation, non cancer-associated fibroblasts, prostate cancer, transcriptomic profiling

## Abstract

**Simple Summary:**

Primary, organ-confined prostate cancer is treatable with surgery, chemotherapy, radiation, and active surveillance; however, a subset of patients will develop metastatic disease. The main issue in prostate cancer is the need of prognostic tools for identifying patients at risk for lethal metastatic tumor, as well as a lack of curative therapies for such patients. The tissues surrounding the prostate cancer cells consist of a mixture of stromal cells that are structurally and functionally different from stromal cells in normal prostate. The stromal component, as a source of prognostic information for metastatic disease progression, has received little attention. In the present study, we characterized stromal cells from cancer tissues as compared to stromal cells from normal adjacent tumor tissue or human benign prostate. The stroma’s specific profile will allow to predict the presence of aggressive tumors, therapy resistance, and metastasis development.

**Abstract:**

Background: Stromal components surrounding epithelial cancer cells seem to play a pivotal role during epithelial-to-mesenchymal transition (EMT), tumor invasion, and metastases. To identify the molecular mechanisms underlying tumor–stroma interactions may yield novel therapeutic targets for prostate cancer. Methods: Gene expression profile of prostate-cancer associated fibroblast (PCAF) and prostate non-cancer associated fibroblast (PNAF) cells isolated from radical prostatectomy was performed by Illumina, analyzed, and further processed by Ingenuity^®^: IPA^®^ software. qRT-PCR was performed on an independent set of 17 PCAF, 12 PNAF, and 12 fibroblast cell lines derived from patients with benign prostatic hyperplasia (BPHF). Results: Using microarray analysis, we found six upregulated genes and two downregulated genes in PCAFs compared to PNAFs. To validate microarray results, we performed qRT-PCR for the most significantly regulated genes involved in the modulation of proliferation and androgen resistance on an independent set of PNAF, PCAF, and BHPF samples. We confirmed the increased expression of SCARB1, MAPK3K1, and TGF-β as well as the decreased expression of S100A10 in PCAFs compared to PNAFs and BPHFs. Conclusions: These results provide strong evidence that the observed changes in the gene expression profile of PCAFs can contribute to functional alteration of adjacent prostate cancer cells.

## 1. Introduction

Primary, organ-confined prostate cancer is treatable with surgery, androgen deprivation therapy, radiation, and active surveillance. However, a subset of patients will develop metastatic disease, with a median survival of less than five years. The main issue in prostate cancer is the need of prognostic tools for identifying patients at risk for lethal metastatic tumors, as well as a lack of curative therapies for such patients. 

In the prostate gland, interactions between epithelium and the stromal microenvironment are required during normal development and to maintain organ function. These interactions provide proliferative and migratory signals that modulate anatomical and positional information. Alterations in these pathways can promote tumorigenesis [1].

The stromal compartment also represents an integral part of the prostate cancer cell community with a main role in cancer progression. Indeed, the tumor microenvironment, through physical contact and the production of several factors and extracellular matrix components, seems to modulate tumor growth, angiogenesis, progression to androgen independence, local invasion, and distant metastasis [2]. Understanding these interactions may contribute to the identification of new diagnostic/prognostic markers and to the development of novel therapies [1]. 

The tissues surrounding the prostate cancer cells consist of a mixture of fibroblasts, myofibroblasts, endothelial cells, immune cells, and extracellular matrix. Stromal cells adjacent to cancer cells are structurally and functionally different from stromal cells in normal prostate. The stromal component, as a source of prognostic information for metastatic disease progression, has received little attention, with most research focusing on tumor cell characteristics [2]. Could the stroma’s specific profile predict the presence of an aggressive tumor, therapy resistance, and metastasis development? Dakhova et al. evaluated global gene expression analysis of reactive stroma in prostate cancer. Several processes, including neurogenesis, axogenesis, and the DNA damage/repair pathways, appeared to be altered in grade 3 reactive stroma surrounding cancer cells compared to normal stroma [3]. However, there is very poor knowledge about the characterization of dysregulated pathways in prostate cancer-associated fibroblasts (PCAFs) and potentially involved in both development and progression of prostate cancer. More research, particularly on matched patient cases, is needed to understand how the stroma of the primary tumor changes during disease stages and metastatic processes. The utilization of computational devices and progressed techniques has empowered the identification of stromal parts related with tumor aggressiveness and progression. As a result, stroma may play a key role in the identification of new biomarkers for cancer treatment.

In the present study, we characterized PCAFs through the identification of differentially expressed genes between PCAFs and prostate non-cancer associated fibroblasts (PNAFs) isolated from normal adjacent tumor tissue or human benign prostate. 

## 2. Materials and Methods

### 2.1. Cell Culture

PNAFs and PCAFs of human prostate were obtained from radical prostatectomy specimens from 29 patients without prior treatment. Eight matched paired samples (tumor and tumor-free areas) were dissected by a trained urologic pathologist by macroscopic examination. For each patient, we collected age, grading, and staging. Confirmation of cancerous and non-cancerous regions in the corresponding samples was identified by standard histological staining of frozen sections of the specimens. Fresh tissue pieces adjacent to the defined area were cut in small pieces and cultivated without any enzymatic dissection in a co-culture system as previously described [4]. To achieve a selective outgrowth of fibroblasts from the patient specimens, co-cultures were grown in DMEM supplemented with 10% FCS, Pen/Strep10 μg/mL at 5% CO_2_ and 37 °C. Cells were identified as epithelial cells/fibroblasts by morphology and subcultivated by Trypsin/EDTA on standard cell culture flasks. As non-cancerous control, we cultivated normal fibroblasts from benign prostate hyperplasia tissue (BPHF) of 12 patients, which have histologically no detectable cancer areas in the prostate using the explant culture system, too. To confirm the nature as fibroblasts, cells were subjected to immunostaining in passage 1 using Cytokeratin 18 (CK 18)/Vimentin/alpha-smooth muscle actin (αSMA) antibodies, respectively. The study was approved by the local ethical review board and all patients gave written informed consent. For all analyses, early passage numbers (3–5) of primary cell cultures were used.

### 2.2. Immunfluorescence Staining

For characterization of the stromal cells, we performed an immunofluorescence staining with stromal cell-specific markers. Fibroblasts were cultivated on chamber slides (Becton Dickinson, BD Biosciences) and, after 70% confluence, cells were fixed with methanol and rinsed with phosphate buffered saline (PBS). After washes, cells were permeabilized with 0.5% Triton X-100 in PBS for 10 min and incubated with monoclonal antibodies against vimentin (1:200 dilution), αSMA (1:200 dilution), or CK 18 (1:100 dilution) for one hour. After washing, cells were incubated with a fluorescence linked secondary antibody (goat anti mouse Cy3), covered with an antifade solution, and visualized using an epifluorescence microscope (Nikon AX70). 

### 2.3. Comparative Genomic Hybridization 

For molecular cytogenetic characterization, DNA was isolated from PNAFs and PCAFs with Qiagen DNA isolation columns (Qiagen, Hilden, Germany). The labeling, hybridization, washing, and detection steps were performed using established protocols. After hybridization, slides were analyzed with a digital image analysis system (MetaSystems ISIS 5.2, Altlussheim, Germany) using an Olympus AX61 microscope equipped with a CCD camera (ProgRes MF, Jenoptik, Jena, Germany).

### 2.4. Microarray Analyis

Gene expression profiles were performed using the Human HT-12 v4.0 BeadChips whole-genome gene expression direct hybridization assay (Illumina, San Diego, CA, USA). This assay is based upon fluorescence detection of biotin-labelled cRNA. Each array contains full-length 50-mer probes representing more than 48,000 well-annotated reference sequence transcripts, including >25,400 unique and up-to-date genes derived from the National Centre for Biotechnology Information Reference Sequence. Total RNA was isolated from 8 PNAF and PCAF samples (matched paired) using the nucleospin RNAII Kit (Machery and Nagel, Dueren, Germany) according to manufacturer’s recommendations. Extracted RNA was stored at −80 °C until further analysis. A total of 300 ng of total RNA was converted to cDNA, followed by an in vitro transcription step to generate biotin-16-UTP-labelled cRNA using the Ambion Illumina Total Prep RNA Amplification Kit (Ambion, Austin, TX, USA) according to the manufacturer’s instructions. The amount and quality of labelled cRNA were measured using the NanoDrop spectrophotometer. The labelled probes were then mixed with hybridization reagents and hybridized overnight on the Human HT-12 v4 BeadChips. After washing and staining, we imaged the BeadChips using the Illumina BeadArray Reader (Illumina) to measure fluorescence intensity for each probe. Using this system, the average signal intensity corresponds to the quantity of respective mRNA in the original sample. Bead summary data were imported into Bead Studio software to remove control probes and to produce a text file containing the signal and detection *p* values for probes for each sample. Text files were imported into the Genome Studio program (Illumina) for statistical analysis. A list of genes reported those that were either upregulated or downregulated to a statistically significant degree. The cut-off used for significance was a *p* value of 0.05.

### 2.5. Reverse Transcription and Real-Time PCR

For validation of the microarray analysis and measuring of the stability of PCAF related genes during cell culturing, total RNA was extracted from PCAFs, PNAFs, and BPHFs in passages 2–7 with RNA Nucleospin RNAII Kit (Machery and Nagel, Dueren, Germany) according to the manufacturer’s instructions. Reverse transcription reactions of 2 µg total RNA were performed with the RNA to cDNA Kit (Applied Biosystems, Foster City, CA, USA). A total of 50 ng cDNA was used in the real-time PCR reactions with gene specific primers using the TaqMan gene expression master mix (Applied Biosystems, Foster City, CA, USA). The run was performed in triplicates using a StepOne Plus real time thermocycler (Applied Biosystems, Foster City, CA, USA). All primers used were purchased by Applied Biosystems (Applied Biosystems, Foster City, CA, USA). Relative expression values were calculated as 2^Δ*C*t^, where Δ*C*_t_ = experimental gene *C*_t_ value—control gene *C*_t_ value. The TATA box binding protein (TBP) served as control. All primers and kits used for the qRT-PCR were purchased by Applied Biosystems: S100 calcium binding protein A16 (S100A10, Assay ID: Hs00293488_m1), mitogen-activated protein kinase kinase kinase (MAP3K1, Assay ID: Hs00394890_m1), scavenger receptor class B 1 (SCARB 1, Assay ID: Hs00969821_m1), Androgen receptor (AR, Assay ID: Hs00171172_m1); skeletal muscle actin, alpha 1, (αSMA, Assay ID: Hs00559403_m1); platelet-derived growth factor receptor, beta (PDGFRβ, Assay ID: Hs01019589_m1); fibroblast activation protein (FAP, Assay ID: Hs00990806_m1); SDF1 or chemokine (C-X-C motif) ligand 12 (Assay ID: Hs00171022_m1); Assay ID: Hs01023894_m1; TGF-β1 (transforming growth factor, beta 1, Assay ID: Hs00998133_m1); TATA box protein binding (Assay ID: Hs00427620_m1). Mean values calculated by relative quantification (2^−ΔΔCt^ method) were assessed for statistical significance with Mann–Whitney test or Wilcoxon matched pairs test using SPSS 19.0.

### 2.6. Ingenuity Pathway Analysis^®^ (IPA)

For each pair of compared samples, we calculated fold change (FC) as follows: FC = log_2_ (XXX vs. YYY). The genes lists filtered for fold change ±1.2 were imported into Ingenuity Pathway Analysis (IPA) software for core analysis (Qiagen-Ingenuity Systems, Redwood, CA, USA). Canonical pathways and interaction networks were identified based on IPA default analysis setting.

### 2.7. Statistical Analysis 

Data were analyzed using SPSS for Windows, Version 12.0 (SPSS Inc., Chicago, IL, USA). For the determination of statistical significance, Student’s *t* and Fisher’s exact tests were used for continuous and categorical variables, respectively. When analyzing data with multiple comparisons, a corrected *p* value with application of the Bonferroni multiple comparison procedure was used. Statistical significance was set to *p* < 0.05. 

## 3. Results

### 3.1. Characterization of Fibroblasts

Summary of the patient characteristics from which we developed fibroblast cultures used for the microarray expression analysis are listed in Table 1. 

**Figure 1:**
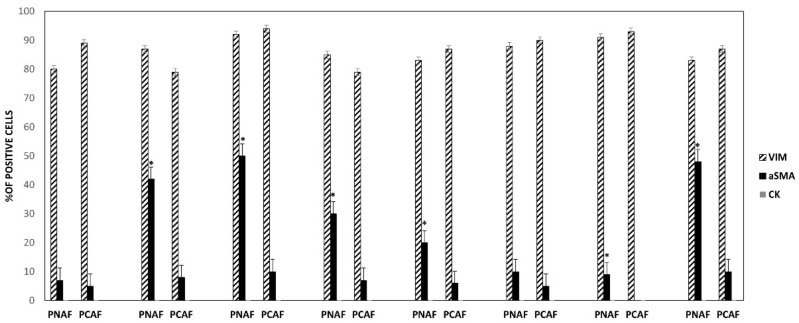
Characterization of patient-derived fibroblast cells. Quantification of the percentage of Vimentin (VIM), cytokeratin 18 (CK), and αSMA positive cells per total number of cells in a given field after immunofluorescence staining of matched paired prostate-cancer-associated fibroblasts (PCAFs) and non-cancer-associated fibroblasts (PNAFs) cell cultures obtained from radical prostatectomy in different patients. * *p* ≤ 0.001.

To characterize the phenotype of stromal cells within the outgrowing cells we performed immune fluorescence staining by focusing on typical fibroblast marker proteins such as vimentin, skeletal muscle actin, alpha 1 (αSMA), and the epithelial marker protein cytokeratin 18 (CK18) in the matched paired samples. The staining reveals a strong vimentin expression in all fibroblasts, whereas CK18 was not detectable. Indeed, we observed differences in the staining pattern of αSMA in PCAFs vs. PNAFs. Positivity of αSMA varied between 5 and 50% in PNAFs and only 5–10% in PCAFs in passages 3 (Figure 1).

For the cytogenetic characterization of the fibroblasts, all cell cultures were assayed by comparative genomic hybridization (CGH) analysis. CGH revealed cytogenetic normal karyotypes without any detectable chromosomal alterations in PNAFs as well as in PCAFs (Table 1). 

### 3.2. Global Expression Profiling of Cancer Associated Fibroblasts and Normal Associated Fibroblasts

To identify genes that were differentially expressed between the matched paired PCAFs and PNAFs, RNA was extracted from each and applied to Illumina HT-12v4.0 Expression BeadChips (Life Technologies, Darmstadt, Germany). Data were analyzed by Genome studio software to identify statistically significant (*p* < 0.05) expression differences and further processed by IPA^®^ software. A total of 21 genes were identified with a statistically significant difference in expression between PCAF and PNAF cells of at least 1.2-fold; among these genes, 16 were upregulated and 5 down-regulated in PCAFs compared to PNAFs (Table 2).

**Figure 2:**
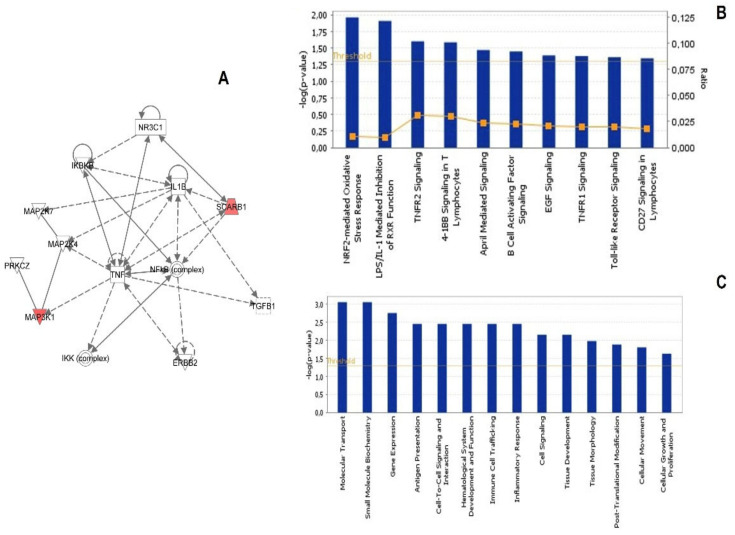
Ingenuity Pathways Analysis (IPA) summary. To investigate possible interactions of differently regulated genes, datasets representing 21 genes with altered expression profile obtained from the Illumina microarray were imported into the Ingenuity Pathway Analysis Tool, and the following data are represented: (**A**) The network analysis of the most highly rated network (cell death, cellular development, and hematopoiesis). Genes shaded in red were determined to be significantly regulated from the statistical analysis and were validated by real-time PCR. A solid line represents a direct interaction between the two gene products and a dotted line means there is an indirect interaction. (**B**) Pathway analysis. The top canonical pathways with *p*-values ranging from 1.01 × 10^−2^ to 3.26 × 10^−2^ are represented. (**C**) Biofunction annotations. The x-axis represents the top biological functions as calculated by IPA based on differentially expressed genes, and the y-axis represents the ratio of number of genes from the dataset that map to the pathway and the number of all known genes ascribed to the pathway. The yellow line represents the threshold of *p* < 0.05 as calculated by Fischer’s test.

IPA functional annotation identified seven top molecules including Magnesium transporter 1 (MAGT1), Mitogen-activated protein kinase kinase kinase 1, E3 ubiquitin protein ligase (MAP3K1), Pleckstrin homology-like domain, family B, member 2 (PHLDB2), RNA Ro-associated Y3 (RNY3), Scavenger receptor class B, member 1 (SCARB1) as up-regulated and S100 calcium binding protein A16 (S100A10) and Small nuclear RNA activating complex, polypeptide 2, (SNAPC2) as down-regulated. The top network is shown in Figure 2A with a score of 4 and with the associated functions of cell death, cellular development, and hematopoiesis. The central node was represented by the Tumor Necrosis Factor (TNF) connected to nuclear factor kappa-light-chain-enhancer of activated B cells (NFkB) complex, Transforming Growth Factor, beta 1 (TGF-β), and Mitogen-Activated Protein Kinase Kinase 7 (MAP2K7) and 4 and MAP3K1. Other molecules directly connected to the central node were Interleukin-1 beta (IL1B), IkB kinase (IKK), Inhibitor of kB (IKB), and Erb-B2 Receptor Tyrosine Kinase 2 (ERBB2). Pathway analysis identified the top canonical pathways illustrated in Figure 2B with *p*-values ranging from 1.01 × 10^−2^ to 3.26 × 10^−2^ for the most significant including Nuclear factor erythroid 2–related factor 2 (NRF-2)-mediated oxidative response, Lipopolysaccharide (LPS)/IL-1-mediated inhibition of Retinoid X receptor (RXR) function, Tumour Necrosis Factor Receptor 1 and 2 signaling, 4-IBB signaling in T-lymphocytes, April mediated signaling, B-cell activating factor signaling, epidermal growth factor (EGF)-signaling, Toll-like receptor signaling, and CD27 signaling in lymphocytes. Biofunction annotation identified, among the most significant, modulations in the following different biological functions: Molecular Transport, Small Molecule Biochemistry, Gene Expression, Inflammatory Response, Cell Signaling, Post-Translational Modification, Cell-to-Cell signaling and interaction, Immune Cell Trafficking (Figure 2C).

### 3.3. Validation of the Microarray Results and Expression of CAF Related Genes in PCAF and Benign Prostate Tissues

**Figure 3:**
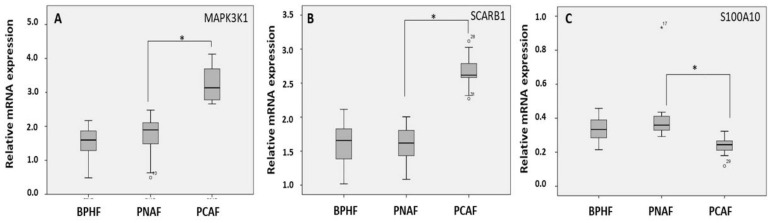
Validation of the deregulated genes derived from microarray analysis. Expression of MAP3K1 (**A**), SCARB1 (**B**), and S100A10 (**C**) expression in PCAF compared to PNAF derived from adjacent tumor tissues and BPHF derived from tumor-free prostate tissues. Gene expression was evaluated by qRT-PCR analysis. The values shown are the 2^−ΔΔCt^ ratios of the difference between cycle thresholds (ΔCt) of the corresponding marker genes and the housekeeping gene TBP; *n* = 3 independent differentiation experiments * *p* ≤ 0.001.

To validate the results of previous microarray analysis, we performed a real time PCR analysis on three highly differentiated genes: MAPK3K1, SCARB1, and S100A10. We selected these genes because their known biological functions indicate that they may play a role in prostate cancer pathogenesis and/or progression. RNA levels were determined by quantitative real-time PCR in fibroblasts derived from 17 prostate carcinoma tissues (PCAFs) and 12 benign tissues from cancer-carrying prostates (PNAFs). As a control, we used fibroblasts from 12 patients without any malignancy in the prostate (BPHFs). mRNA levels of MAP3K1 and SCARB1 were found to be significantly increased in the cancerous versus benign tissues, whereas S100A10 levels were decreased. Therefore, qRT-PCR confirmed results derived from microarray analysis (Figure 3A–C).

**Figure 4:**
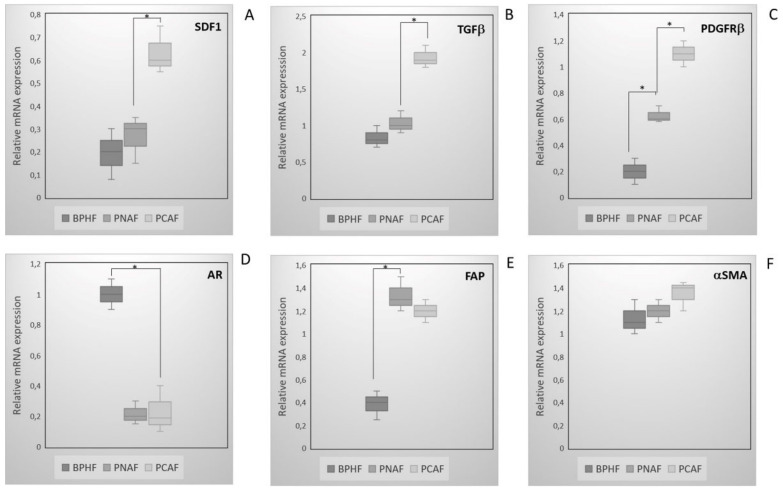
Expression of CAF-related genes in an independent set of PCAF, PNAF, and BHPF samples. Expression levels of SDF1 (**A**), TGFβ (**B**), PDGFRβ (**C**), AR (**D**), FAP (**E**), and αSMA (**F**) were detected in 17 PCAFs compared to 12 PNAFs and 12 BPHFs by qRT-PCR analysis. The values shown are the 2^−ΔΔCt^ ratios of the difference between cycle thresholds (ΔCt) of the corresponding marker genes and the housekeeping gene TBP; *n* = 3 independent differentiation experiments * *p* ≤ 0.001.

Additionally, we measured mRNA levels of the cancer-associated fibroblastrelated genes described in the literature such as αSMA, or those resulting from IPA analysis including stromal-derived factor 1 (SDF1), TGF- β, platelet-derived growth factor receptor β (PDGFRβ), and fibroblast activating protein (FAP) in PCAFs, PNAFs, and BPHFs (Figure 4). While expression of SDF1 and TGF-β genes was significantly stronger in PCAFs compared to PNAFs and BPHFs, no significant differences were observed among the expression of Androgen Receptor (AR), FAP, and αSMA in PCAFs and PNAFs (Figure 4A,B,D–F). Quantitative RT-PCR revealed PDFGRβ mRNA to be generally overexpressed in prostate cancer fibroblasts independently from their topological localization (i.e., PNAF/PCAF) compared to benign samples (Figure 4C). The expression of AR and FAP was significantly different in BPHFs versus PNAFs. Interestingly, levels of AR mRNAs were dramatically higher in BPHF compared to PCAF and PNAF (Figure 4D). Expression levels of FAP were similar in PCAFs and PNAFs but significant lower in BPHFs (Figure 4E). αSMA mRNA expression was similar between all three tissues (Figure 4F).

**Figure 5:**
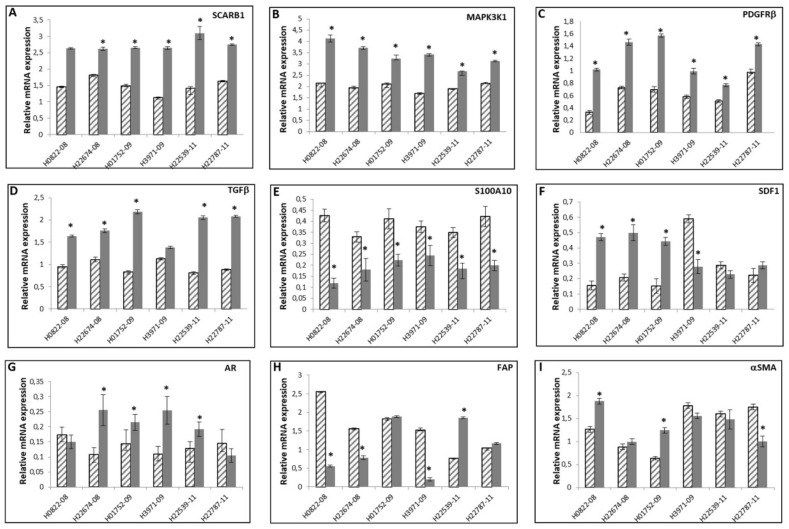
Expression of CAF-related genes in matched paired cancer and non-cancer associated fibroblasts. Expression levels of SCARB1 (**A**), MAPK3K1 (**B**), S100A10 (**C**), PDGFRb (**D**), TGFb (**E**), SDF1 (**F**), AR (**G**), FAP (**H**), αSMA (**I**) in PNAF (striped bars) and PCAF (gray bars) derived from six different prostate cancer patients (matched samples) were evaluated by RT-PCR. The values shown are the 2^−ΔΔCt^ ratios of the difference between cycle thresholds (ΔCt) of the corresponding marker genes and the housekeeping gene TBP; *n* = 3 independent differentiation experiments * *p* ≤ 0.001.

In the next step, we compared the expression levels of the selected genes in matched paired samples of PCAFs and PNAFs derived from six different patients (Figure 5A–I). Expression levels of SCARB1, MAPK3K1, PDGFRβ, and TGF-β were in all cases increased, and S100A10 mRNA decreased in PCAFs compared to PNAFs (Figure 5A–E). The expression of the CAF-related marker genes SDF1, FAP, αSMA, and AR for activated phenotype of cancer associated fibroblasts were inconsistently deregulated in the six analyzed paired samples (Figure 5F–I).

In addition, we performed a correlation analysis between the three highly deregulated genes derived from microarray analysis and both clinical pathological characteristics and patient outcomes. Interestingly, bioinformatic analysis based on the TGCA dataset showed that SCARB1 expression was significantly increased in prostate cancer samples compared to normal tissues while MAPK3K1 and S100A10 expression was significantly reduced. Moreover, SCARB1 expression positively correlated with nodal metastasis status and Gleason score while MAPK3K1 and S100A10 levels inversely correlated. The expression and clinical correlation analysis via the UALCAN database revealed that the transcription levels of SCARB1 were significantly increased according to Gleason score. Compared with the patients in the Gleason score 6–9 groups, SCARB1 displayed the highest transcription levels in the Gleason score 10 group. The positive correlation between SCARB1 expression and Gleason score might indicate a promoting role of SCARB1 signaling in PCa. On the other hand, we found that S100A10 and MAPK3K1 expression was reduced in higher Gleason score groups but compared with the patients in the Gleason score 6–9 groups, S100A10 levels increased in the Gleason score 10 group (Figure S1). Furthermore, to evaluate the role of the expression of these three gene in the prognosis of PCa patients, we used GEPIA to assess the correlation between SCARB1, MAPK3K1, and S100A10 expression and clinical outcomes. A survival map showed that SCARB1, MAPK3K1, and S100A10 expression may affect prognosis in PCa patients. In details, the overall survival (OS) analysis implied that OS was significantly higher in low SCARB1 than in high SCARB1 group. On the other hand, high MAPK3K1 and S100A10 positively influenced PCa patient OS (Figure S1).

### 3.4. Stability of the Activated Stromal Phenotype during Cell Culturing 

**Figure 6:**
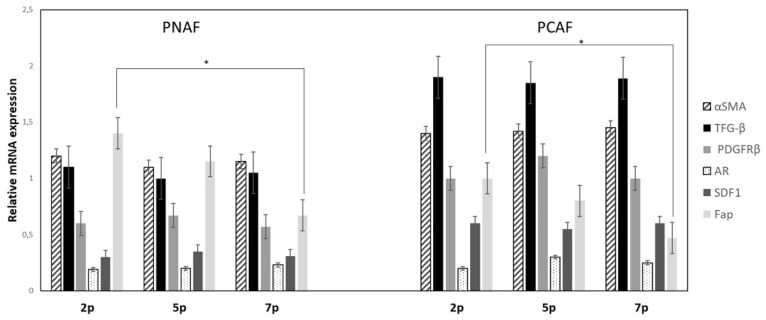
CAF-related gene expression during cell culturing. Expression of αSMA, TGFb, PDGFRb, AR, SDF1, and FAP on total RNA isolated from PCAFs and PNAFs during culture conditions from passage 2 to passage 5 and 7 was evaluated by RT-qPCR. The values shown are the 2^−ΔΔCt^ ratios of the difference between cycle thresholds (ΔCt) of the corresponding marker genes and the housekeeping gene GAPDH; *n* = 3 independent differentiation experiments * *p* ≤ 0.001.

To evaluate whether cell culture effects take place in fibroblast cultures over several passages and after how many cell culture passages the activated stromal phenotype is stable, we performed a qRT-PCR analysis for up to seven passages. For this approach, we evaluated the expression levels of PCAF related genes like αSMA, SDF1, TGF-β, PDGFRβ, and FAP in PCAF and PNAF cell lines derived from two different patients from passage two to seven. All mRNA expression levels were measured in comparison to a calibrator mRNA derived from several cell lines (mRNA = 1.0). We detected a constant expression of αSMA, TFG-β, PDGFRβ, AR, and SDF1 during culturing whereas expression levels of the FAP gene dramatically decreased from p2 to p7 (Figure 6).

## 4. Discussion

Activation of the host stromal microenvironment is a key player in the initiation and progression of prostate cancer [5]. Reactive stroma consists of a mixture of fibroblasts, myofibroblasts, vasculature-related cells (endothelial cells, pericytes, and smooth muscle cells), and immune cells (lymphocytes, macrophages, and mast cells). Several growth factors produced by cancer cells are able to activate stromal compartment through a paracrine manner. On the other hand, these modified stromal cells secrete several soluble factors and extracellular matrix proteins, which play an important role in cancer development [5,6].

Therefore, in a process that resembles the wound healing pathway, the host stromal component reacts to carcinoma to maintain tissue homeostasis. Through such interactions, the stromal cells acquire an activated phenotype and function. This new stromal microenvironment promotes tumor invasion and metastasis by supporting cancer cell survival, proliferation, and migration, and by inducing angiogenesis [6].

There is a considerable interest in identifying the molecular mechanisms underlying tumour–stroma interactions. Indeed, this may yield novel therapeutic targets for prostate cancer, especially for androgen-independent and metastatic diseases [7].

Few studies have examined the transcriptional profile of cancer stroma and stromal subsets [3,6,8,9] to identify genes potentially involved in prostate cancer induction and progression.

The first transcriptome analysis of laser-captured stroma from matched normal and tumor areas evidenced 44 dysregulated transcripts in the intratumoral stroma, involving a large number of pathways [8]. Zhao et al. compared the gene expression profiles of human stromal cell cultures from normal transition zone, normal peripheral zone, benign prostatic hyperplasia, and prostate cancer. Many of the differentially expressed genes were involved in biological processes known to be important in the development of prostatic diseases including cell proliferation and apoptosis, cell adhesion, and immune response [9].

Dakhova et al. evaluated global changes in gene expression in prostate cancer reactive stroma grade 3 relative to paired benign prostatic stroma using microarray analysis on laser captured RNAs from these two tissue types [3]. A total of 544 unique genes were higher in the reactive stroma and 606 unique genes were lower based on microarray analysis compared to benign stroma. The upregulated genes were associated with a variety of biological processes including stem cell maintenance, axonogenesis/neurogenesis, angiogenesis, and alterations of extracellular matrix [3]. Among stromal host cells, activated fibroblasts are demonstrated to be mainly involved in the growth and dissemination of prostate cancer. Reinertsen et al. compared the gene expression profile of PCAFs with that of fibroblasts from hyperplastic areas. Several genes involved in cell proliferation, angiogenesis, inflammatory response, and cell adhesion were differentially expressed between the two types of fibroblasts [6]. 

Orr et al. examined the transcripts expressed in patient-matched pair of PCAF, PNAF cells, and fetal prostate using Tag profiling. Gene ontology analysis revealed that PCAF-enriched transcripts were associated with prostate morphogenesis and PCAF-depleted transcripts were associated with cell cycle [10].

Many CAF-related markers have been investigated but only a few of them have been translated into clinical practice, probably because of the widespread heterogeneity of CAFs [11,12]. Indeed, CAFs may derive from different cell types, including fibrocytes, endothelial cells, mesenchymal stem cells, and stellate cells. Recent studies have identified several CAF subclusters with specific signatures and different response to therapies, therefore, internal heterogeneity in CAF subpopulations represents a new challenge in medicine [12]. In this light, novel technologies such as single-cell sequencing and mass spectrometry have been developed to study single-cell expression profiles and identify new biomarkers for cancer since CAF-derived proteins represent the main actors in intercellular crosstalk [13,14,15].

In our study, for the first time, we have identified increased expression of SCARB1 and MAPK3K1 and decreased expression of S100A10 in PCAFs compared to PNAFs even if the role of these genes in prostate cancer progression and androgen resistance is well known. 

We assessed the gene expression profile of PCAFs and their normal paired counterparts in prostate cancer samples in order to identify: (i) relevant pathways responsible for pro-tumorigenic effects; (ii) new biomarkers for cancer treatment focused on the stroma. To identify genetic changes that occur when PNAFs from adjacent tissues are transformed into PCAFs could allow to clarify pathways that play a key role in the crosstalk between fibroblasts and tumor cells. Such results may provide significant biomarkers for future target discovery or tumor classification. Cancer-associated fibroblasts (CAFs) influenced tumor development through different mechanisms. Several studies have reported that CAF-mediated immunological modulation, angiogenesis, and metabolic reprogramming of tumor microenvironment have been implicated in cancer cell survival [2,16]. Using six paired PNAF-PCAF samples, we identified a subset of 21 genes that were differentially expressed between the two fibroblast populations. The purity of isolated fibroblasts was confirmed by detecting specific fibroblast biomarkers. These cells expressed vimentin and αSMA, whereas they were negative for CK 18 (epithelial marker). In details, we identified 16 up-regulated genes and 5 down-regulated genes in PCAFs compared to PNAFs. IPA functional annotation identified seven top molecules including MAGT1, MAP3K1, PHLDB2, RNY3, and SCARB1 as up-regulated and S100A10 and SNAPC2 as down-regulated. Differentially expressed genes were linked to different biological functions as well as Molecular Transport, Small Molecule Biochemistry, Gene Expression, Inflammatory Response, Cell Signaling, and Post-Translational Modification. Our findings demonstrated that stromal PCAFs may regulate tumor progression and recurrence through the TNF family pathway connected to NFkB, TGF-β, and MAP kinases. Other molecules directly connected to the central node were several pro-inflammatory molecules IL1B, IKK, IKB, and ERBB2. Oncogenic TGF-β signaling has been linked to PCa; nevertheless, a signature of TGF-β-regulated ECM genes has been identified in many CAFs and associated with poor prognosis [17,18,19].

TGF-β signaling plays a key role in CAFs as demonstrated in more detail in breast cancer models; in fact, TGF-β and SDF1 take part in two autocrine and cross-talking signaling loops that are involved in myofibroblast/CAF transition [20,21]. High levels of TGF-β target SNAI1 in fibroblasts induce an increase of SDF-1 secretion [22]. CAFs secrete TGF-β and SDF-1 by promoting angiogenesis and stimulating cell proliferation and EMT [23]. Simultaneously, in myofibroblasts autocrine TGF-β/SDF-1 pathway activates SDF-1 signaling. Thus, once triggered, this positive feedback loop supports myofibroblast differentiation and tumor progression by targeting endothelial and tumor cells [24].

To validate the results of our preceding microarray analysis, we performed a real time PCR analysis of three highly differentiated genes MAP3K1, SCARB1, and S100A10. We selected these genes because their known biological functions indicate that they play a role in prostate cancer pathogenesis and/or progression [25,26,27]. MAP3K1 encodes a MEK kinase involved in the regulation of cell proliferation, SCARB1 plays an important role in the regulation of cell homeostasis by maintaining the level of cholesterol available in fibroblasts for androgen synthesis, and S100A10 controls migration of cancer-associated macrophages [25,26,27,28,29,30,31,32,33].

mRNA levels of MAP3K1 and SCARB1 were found to be significantly increased in the cancerous versus benign tissues, whereas S100A10 levels were decreased. Therefore, qRT-PCR confirmed results derived from microarray analysis in an independent cohort of samples. 

Additionally, we measured mRNA levels of the cancer-associated fibroblast-related genes described in the literature such as αSMA [34], or that resulted from IPA analysis including SDF1, TGF-β, PDGFRβ, and FAP [34] in an independent cohort of PCAFs, PNAFs, and BHPFs derived from patients. Although expression of the SDF1 and TGF-β genes was significantly stronger in PCAFs compared to PNAFs and BPHFs, no significant differences were observed among the expression of AR, FAP, and αSMA in PCAFs and PNAFs. Interestingly, levels of AR mRNAs were higher, whereas FAP and PDGFR levels were lower in BPHF compared to PCAFs and PNAFs. αSMA mRNA expression was similar between all three tissues. 

Thereafter, we compared the expression levels of the selected genes in matched paired samples of PCAFs and PNAFs derived from six different patients. Expression levels of SCARB1, MAPK3K1, PDGFRβ, and TGFβ were in all cases increased, and S100A10 mRNA decreased in PCAF compared to PNAF. The expression of the CAF-related marker genes for activated phenotype for cancer-associated fibroblast SDF1, FAP, αSMA, and the androgen receptor were inconsistently deregulated in the six analyzed paired samples.

Classical genes associated with an activated status of fibroblasts (FAP, PDGFRβ) [24] were higher in normal adjacent tissues compared with normal tissues from healthy patients whereas AR levels that work as transcriptional repressors of cancer-associated fibroblast activation were lower. Such validation proves that adjacent tumor tissue already has genetic changes that are enhanced in PCAFs. We hypothesize that it could be a result of paracrine crosstalk with tumor cells to create an adequate niche to increase tumor size and invade. 

Prostate cancer show a wide heterogeneity in protein expression. Recently, novel biomarkers that are associated with diagnosis, prognosis, and are related to cell proliferation have been identified [35,36]. Our aim was to demonstrate that stroma’s profile could predict the risk to develop prostate cancer and its prognosis, since some molecules secreted by CAFs may induce prostate cancer progression; on these bases, we performed a correlation analysis between SCARB1, MAPK3K1, and S100A10 expression and both clinical pathological characteristics and patient outcomes. We found that SCARB1 expression positively correlated with prostate cancer, nodal metastasis status, and Gleason score while MAPK3K1 and S100A10 levels inversely correlated. Furthermore, low SCARB1 expression and high MAPK3K1 and S100A10 levels positively affected the OS of PCa patients. Several studies have demonstrated a lack of concordance between the Gleason score (GS) derived from the prostate biopsy and the radical prostatectomy. Recently, a meta-analysis performed on 14,839 patients showed that 30% of the patients underwent to GS upgrade, and about 63% remained unmodified after prostatectomy. Gleason upgrade resulted worse in the sub-group of patients with a biopsy GS 3 + 3 [37]. Our findings might identify promising candidate markers for selecting biopsy GS 6 prostate cancer at risk for up-grading at prostatectomy. 

Finally, to monitor the stability of gene expression profile during cell culture we quantified the expression levels of some CAFs specific genes like FAP, αSMA, PDGFRβ, and TGF-β during culture conditions. We detected a constant expression of αSMA, TFG-β, PDGFRβ, AR, and SDF1 during culturing whereas expression levels of the FAP gene significantly decreased from passage 2 to passage 7. All our analysis were performed on fibroblast cell lines at passage 3 to avoid any interference.

## 5. Conclusions

This represents a comprehensive gene expression analysis of PCAFs, PNAFs, and BPHFs in prostate cancer. For the first time, we have identified increased expression of SCARB1 and MAPK3K1, and decreased expression of S100A10 in PCAFs compared to PNAFs.

We found differentially expressed genes that play a role in prostate cancer pathogenesis and/or progression. Thus, our results provide strong evidence that the observed changes in the gene expression profile of PCAFs can contribute to functional alteration of adjacent prostate cancer cells.

## Figures and Tables

**Table 1:** Clinical-pathological characteristics of the patients.

Case	Age	G1		G2	G Sum	T	CGH Results
	PCAF and PNAF patients (matched samples)						
H01752-09	47	3		3	6	pT2c	46, XY Normal
H03971-09	67	4		3	7	pT2c	46, XY Norma
H08222-08	51	3		3	6	pT2a	46, XY Normal
H14467-08	59	3		3	6	pT2c	46, XY Normal
H19483-08	67	4		4	8	pT3b	46, XY Normal
H22539-11	53	4		5	9	pT3b	46, XY Normal
H22674-08	62	4		3	7	pT2c	46, XY Normal
H22787-11	48	3		4	7	pT2c	46, XY Normal
	PCAF patients						
H00524-06	73	4		3	7	pT3b	46, XY Normal
H01050-06	63	3		4	7	pT2c	46, XY Normal
H01915-09	65	4		3	7	pT3b	46, XY Normal
H03806-11	55	3		3	6	pT2c	46, XY Normal
H04149-05	74	3		4	7	pT3	46, XY Normal
H06123-11	53	3		3	6	pT2a	46, XY Normal
H06530-11	59	3		4	7	pT3b	46, XY Normal
H06968-09	60	4		3	7	pT3b	46, XY Normal
H06976-08	67	4		3	7	pT3a	46, XY Normal
H07029-11	57	3		4	7	pT3a	46, XY Normal
H07113-09	72	4		3	7	pT3a	46, XY Normal
H07147-11	41	3		4	7	pT2c	46, XY Normal
H10729-05	63	3		4	7	pT3a	46, XY Normal
H11947-08	66	4		3	7	pT3b	46, XY Normal
H14912-09	61	3		4	7	pT3a	46, XY Normal
H14987-09	57	3		4	7	pT2c	46, XY Normal
H15991-05	70	2		3	5	pT2a	46, XY Normal
	PNAF patients						
H02281-09	68	4		3	7	pT3a	46, XY Normal
H02840-05	63	3		4	7	pT2a	46, XY Normal
H03382-09	61	4		4	8	pT2a	46, XY Normal
H03503-09	72	3		4	7	pT2c	46, XY Normal
H07329-08	48	3		4	7	pT2b	46, XY Normal
H10343-07	68	3		3	6	pT2c	46, XY Normal
H11061-05	70	3		4	7	pT3a	46, XY Normal
H19602-05	60	3		4	7	pT2c	46, XY Normal
H19647-08	64	5		4	9	pT3b	46, XY Normal
H21352-05	72	3		4	7	pT2c	46, XY Normal
H24024-08	56	4		3	7	pT3a	46, XY Normal
H07102-05	69	3		4	7	pT2c	46, XY Normal
	BPHF patients						
H06323-08	68	N.A.		N.A.	N.A.	N.A.	46, XY Normal
H08269-08	75	N.A.		N.A.	N.A.	N.A.	46, XY Normal
H03124-08	74	N.A.		N.A.	N.A.	N.A.	46, XY Normal
H14588-08	76	N.A.		N.A.	N.A.	N.A.	46, XY Normal
H01753-09	77	N.A.		N.A.	N.A.	N.A.	46, XY Normal
H03991-09	62	N.A.		N.A.	N.A.	N.A.	46, XY Normal
H00485-13	64	N.A.		N.A.	N.A.	N.A.	46, XY Normal
H00425-13	76	N.A.		N.A.	N.A.	N.A.	46, XY Normal
H06884-13	80	N.A.		N.A.	N.A.	N.A.	46, XY Normal
H08417-13	66	N.A.		N.A.	N.A.	N.A.	46, XY Normal
H11630-13	63	N.A.		N.A.	N.A.	N.A.	46, XY Normal
H23201-13	70	N.A.		N.A.	N.A.	N.A.	46, XY Normal

PCAF: prostate cancer-associated fibroblasts; PNAF: prostate non-cancer-associated fibroblasts; BPHF: benign prostatic hyperplasia fibroblasts; G1: Gleason grade 1; G2: Gleason grade 2; G Sum: Gleason score sum; T: Tumor stage; CGH: Comparative genomic hybridization.

**Table 2:** Significantly modulated genes in cancer associated fibroblasts (*p* < 0.05, FC ± 1.5).

Fold Change	Symbol	Entrez Gene Name	Type(s)	Cell Compartment
1.597	*CLHC1*	clathrin heavy chain linker domain containing 1	other	Other
2.100	*CLK4*	CDC-like kinase 4	kinase	Nucleus
1.496	*FLJ44124*	uncharacterized LOC641737	other	Other
1.462	*HIATL2*	Hippocampus abundant transcript-like 2	other	Other
1.460	*KIAA1598*	KIAA1598	other	Plasma Membrane
1.459	*LOC401098*	uncharacterized LOC401098	other	Other
1.556	*LRRC37BP1*	leucine rich repeat containing 37B pseudogene 1	other	Other
1.456	*MAGT1*	magnesium transporter 1	enzyme	Plasma Membrane
1.816	*MAP3K1*	mitogen-activated protein kinase kinase kinase 1, E3 ubiquitin protein ligase	kinase	Cytoplasm
1.237	*NBPF10* (*includes others*)	neuroblastoma breakpoint family, member 15	other	Other
1.510	*PHLDB2*	pleckstrin homology-like domain, family B, member 2	other	Cytoplasm
1.719	*RNY3*	RNA, Ro-associated Y3	other	Other
2.308	*TXLNGY*	taxilin gamma pseudogene, Y-linked	other	Other
1.499	*ZNF700*	zinc finger protein 700	other	Extracellular Space
1.565	*SCARB1*	scavenger receptor class B, member 1	transporter	Plasma Membrane
1.620	*SMCR5*	Smith-Magenis syndrome chromosome region, candidate 5	other	Other
−1.589	*S100A10*	S100 calcium binding protein A10	other	Cytoplasm
−1.853	*S100A16*	S100 calcium binding protein A16	other	Nucleus
−1.633	*ANXA2P3*	annexin A2 pseudogene 3	other	Other
−1.541	*SNAPC2*	small nuclear RNA activating complex, polypeptide 2, 45kDa	transcription regulator	Nucleus
−1.620	*SPRED2*	sprouty-related, EVH1 domain containing 2	cytokine	Extracellular Space

## Data Availability

Data available in a publicly accessible repository.

## Supplementary Materials

The following supporting information can be downloaded at: https://www.mdpi.com/article/10.3390/cancers14122943/s1, Figure S1: Correlation analysis between MAPK3K1, SCARB1 and S100A10 expressions and clinical pathological characteristics or patient outcomes.
