# Supplementary material for: Molecular Characterization of Cancer Associated Fibroblasts in Prostate Cancer
Source: Cancers (Basel). 2022 Jun 14;14(12):2943. doi: 10.3390/cancers14122943 (PMC9221001; doi:10.3390/cancers14122943)
Supplement: Supplementary file 1 [file cancers-14-02943-s001.zip › cancers-1648744-supplementary.pdf]

cancers

MDPI

Supplementary Materials

Molecular Characterization of Cancer Associated Fibroblasts in Prostate Cancer

Giovanni Vitale [1,2,†], Michele Caraglia [3,†], Volker Jung [4], Jörn Kamradt [4], Davide Gentilini [5,6], Maria Teresa Di Martino [7], Alessandra Dicitore [1], Marianna Abate [3], Pierosandro Tagliaferri [7], Annalisa Itro [3], Matteo Ferro [8], Raffaele Balsamo [9], Marco De Sio [10], Gaetano Facchini [11], Luca Persani [1,12], Kai Schmitt [13], Matthias Saar [4], Michael Stöckle [4], Gerhard Unteregger [4] and Silvia Zappavigna [3,*]

1 Department of Medical Biotechnology and Translational Medicine (BIOMETRA), University of Milan, 20133 Milan, Italy; giovanni.vitale@unimi.it (G.V.); alessandra.dicitore@unimi.it (A.D.); luca.persani@unimi.it (L.P.)
2 Laboratory of Geriatric and Oncologic Neuroendocrinology Research, Istituto Auxologico Italiano (IRCCS), Cusano Milanino, 20095 Milan, Italy
3 Department of Precision Medicine, University of Campania "L Vanvitelli", 80138 Naples, Italy; michele.caraglia@unicampania.it (M.C.); marianna.abate@unicampania.it (M.A.); annalisa.itro@unicapania.it (A.I.)
4 Clinic of Urology and Pediatric Urology, University of Saarland, 66421 Homburg, Germany; volker.jung@uks.eu (V.J.); joern.kamradt@hirslanden.ch (J.K.); matthias.saar@uks.eu (M.S.); michael.stoeckle@uks.eu (M.S.); gerhard.unteregger@uks.eu (G.U.)
5 Bioinformatics and Statistical Genomics Unit, Istituto Auxologico Italiano (IRCCS), 20095 Milan, Italy; davide.gentilini@unipv.it
6 Department of Brain and Behavioral Sciences, University of Pavia, 27100 Pavia, Italy
7 Department of Experimental and Clinical Medicine, University "Magna Graecia" of Catanzaro, 88100 Catanzaro, Italy; teresadm@unicz.it (M.T.D.M.); tagliaferri@unicz.it (P.T.)
8 Division of Urology, European Institute of Oncology-IRCCS, 20132 Milan, Italy; matteo.ferro@ieo.it
9 Urology Clinic, Monaldi Hospital, 80125 Naples, Italy; raffaele.balsamo@unicampania.it
10 Urology Unit, University of Campania "Luigi Vanvitelli", 80138 Naples, Italy; marco.desio@unicampania.it
11 UOC of Medical Oncology, ASL NA 2 Nord, "S.M. delle Grazie" Hospital, 80078 Pozzuoli, Italy; ga.facchi@libero.it
12 Laboratory of Endocrine and Metabolic Research, Istituto Auxologico Italiano (IRCCS), 20095 Milan, Italy
13 Department of Pathology, Saarland University Medical Center, 66421 Homburg, Germany; kai.schmitt@uks.eu
* Correspondence: silvia.zappavigna@unicampania.it; Tel.: +39-081-566-7629
† These authors equally contributed to the manuscript.

A

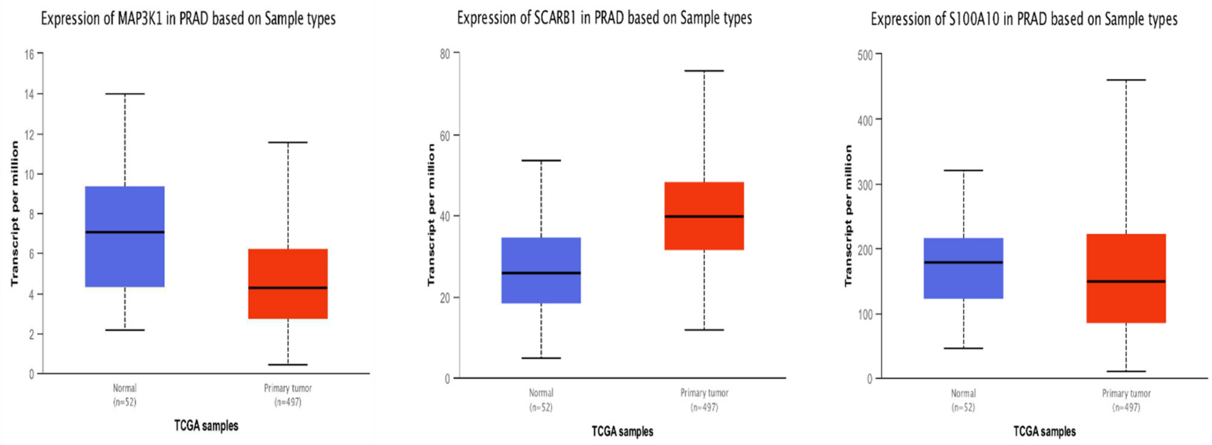

Comparison	Statistical significance
Normal-vs-Primary	2.08489999999895E-05

Comparison	Statistical significance
Normal-vs-Primary	4.98880048382944E-10

Comparison	Statistical significance
Normal-vs-Primary	4.833600E-01

B

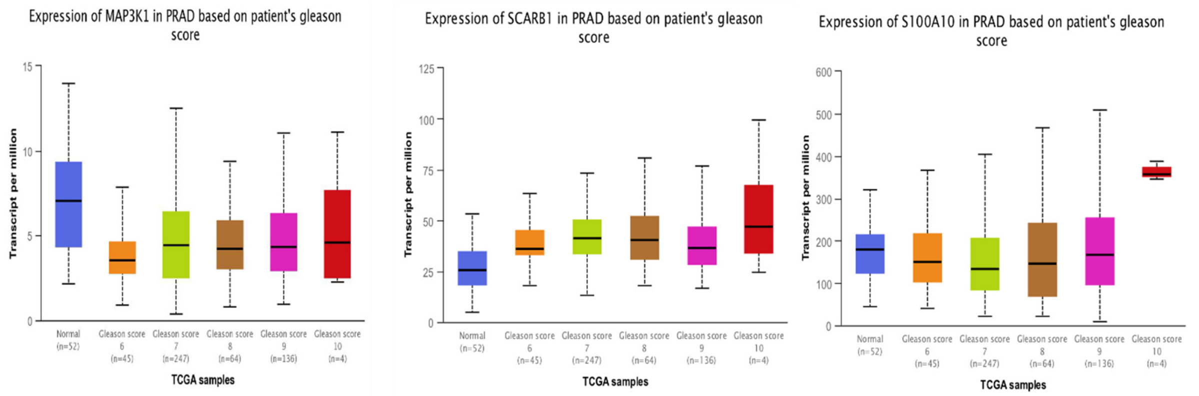

Comparison	Statistical significance
Normal-vs-Gleason score 6	1.26030000002775E-06
Normal-vs-Gleason score 7	9.87139999999442E-05
Normal-vs-Gleason score 8	9.90429999999654E-05
Normal-vs-Gleason score 9	4.1581999999929E-06
Normal-vs-Gleason score 10	3.469200E-01
Gleason score 6-vs-Gleason score 7	3.769900E-02
Gleason score 6-vs-Gleason score 8	1.643150E-01
Gleason score 6-vs-Gleason score 9	3.118400E-01
Gleason score 6-vs-Gleason score 10	5.562600E-01
Gleason score 7-vs-Gleason score 8	6.928200E-01
Gleason score 7-vs-Gleason score 9	1.468150E-01
Gleason score 7-vs-Gleason score 10	7.487800E-01
Gleason score 8-vs-Gleason score 9	4.621000E-01
Gleason score 8-vs-Gleason score 10	6.165600E-01
Gleason score 9-vs-Gleason score 10	4.196600E-01

Comparison	Statistical significance
Normal-vs-Gleason score 6	1.817720E-04
Normal-vs-Gleason score 7	1.1518563880486E-12
Normal-vs-Gleason score 8	1.82429999995293E-07
Normal-vs-Gleason score 9	1.34929999950906E-07
Normal-vs-Gleason score 10	2.009000E-01
Gleason score 6-vs-Gleason score 7	1.122840E-01
Gleason score 6-vs-Gleason score 8	1.907240E-01
Gleason score 6-vs-Gleason score 9	5.191400E-01
Gleason score 6-vs-Gleason score 10	4.230400E-01
Gleason score 7-vs-Gleason score 8	7.706000E-01
Gleason score 7-vs-Gleason score 9	4.267000E-01
Gleason score 7-vs-Gleason score 10	5.261000E-01
Gleason score 8-vs-Gleason score 9	4.466600E-01
Gleason score 8-vs-Gleason score 10	5.512800E-01
Gleason score 9-vs-Gleason score 10	1.933230E-01

Comparison	Statistical significance
Normal-vs-Gleason score 6	2.452200E-01
Normal-vs-Gleason score 7	4.096400E-01
Normal-vs-Gleason score 8	7.738000E-01
Normal-vs-Gleason score 9	2.538100E-02
Normal-vs-Gleason score 10	4.002200E-02
Gleason score 6-vs-Gleason score 7	5.612600E-01
Gleason score 6-vs-Gleason score 8	5.147600E-01
Gleason score 6-vs-Gleason score 9	3.518700E-03
Gleason score 6-vs-Gleason score 10	1.93400000014776E-07
Gleason score 7-vs-Gleason score 8	7.602800E-01
Gleason score 7-vs-Gleason score 9	4.519900E-03
Gleason score 7-vs-Gleason score 10	3.11409999997903E-06
Gleason score 8-vs-Gleason score 9	2.886200E-02
Gleason score 8-vs-Gleason score 10	2.776500E-04
Gleason score 9-vs-Gleason score 10	9.831800E-02

C

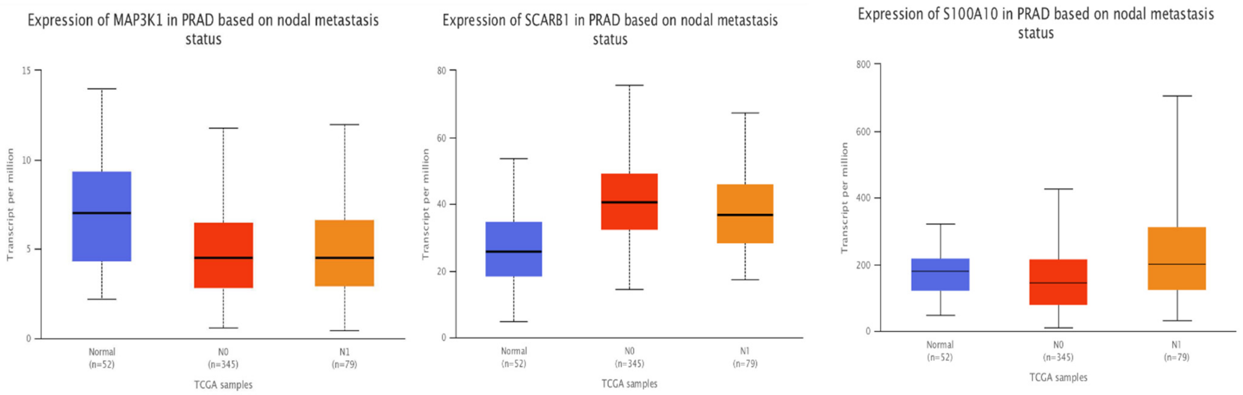

Comparison	Statistical significance
Normal-vs-N0	8.25110000000073E-05
Normal-vs-N1	1.248830E-04
N0-vs-N1	8.083200E-01

Comparison	Statistical significance
Normal-vs-N0	3.38209460437611E-11
Normal-vs-N1	1.69449999998239E-06
N0-vs-N1	9.552000E-01

Comparison	Statistical significance
Normal-vs-N0	8.565800E-01
Normal-vs-N1	6.624400E-03
N0-vs-N1	4.104600E-03

D

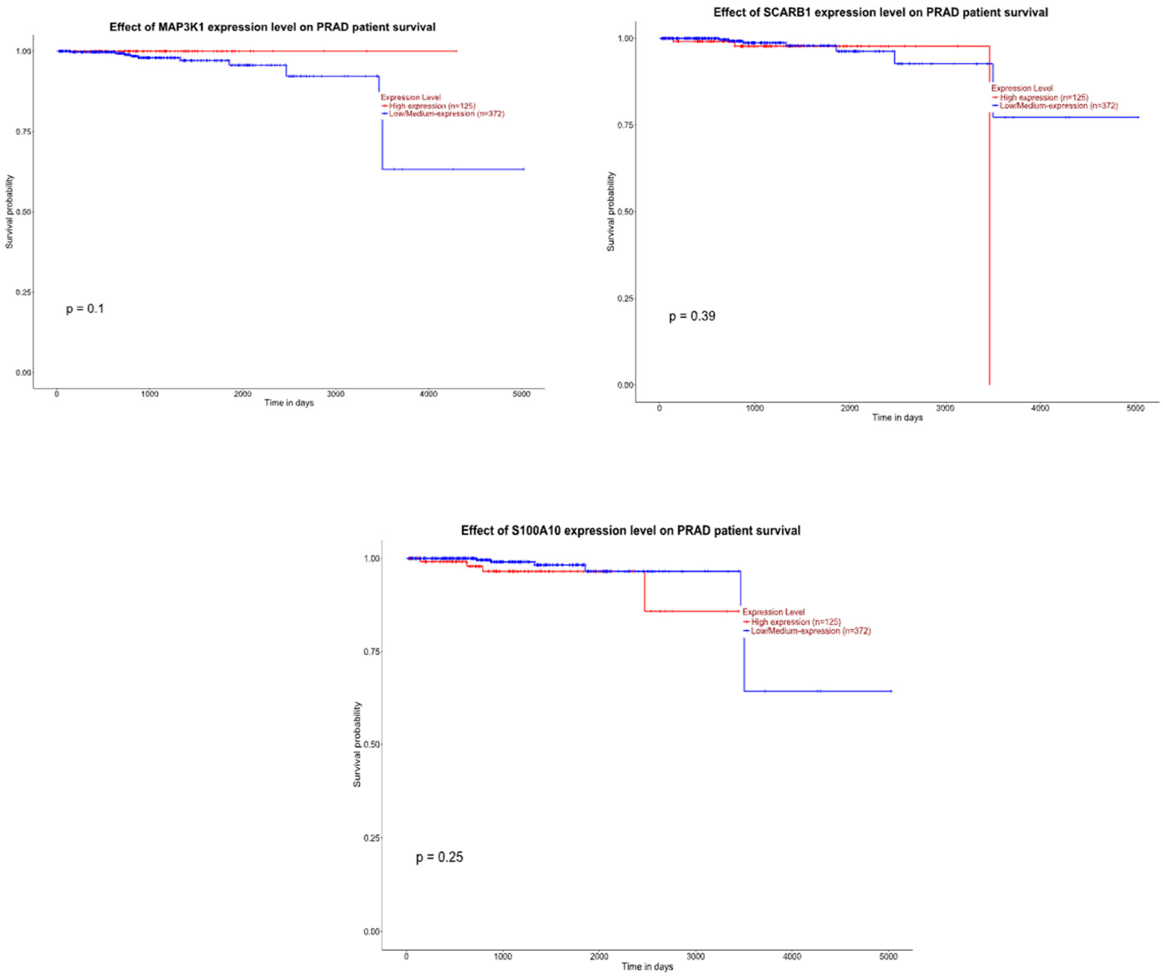

Figure S1 legend

Figure S1. Correlation analysis between MAPK3K1, SCARB1 and S100A10 expressions and clinical pathological characteristics or patient outcomes. (A) TCGA-based UALCAN database screening and analysis showing a significant difference in the MAPK3K1, SCARB1 and S100A10 expressions between normal tissue and PCa tissue (B) UALCAN database analysis showing that the transcriptional level of MAPK3K1, SCARB1 and S100A10 significantly correlated with increasing Gleason score.(C) UALCAN database analysis showing that the transcriptional level of MAPK3K1, SCARB1 and S100A10 significantly correlated with nodal metastasis status. (D) Cox regression analysis showing that patients in the low SCARB1 groups and in the high MAPK3K1 and S100A10 groups had a significant better overall survival (OS).

Bioinformatic Analysis

Gene Expression Profiling Interacting Analysis (GEPIA2; http://gepia2.cancer-pku.cn/) was used to analyze the RNA sequencing expression data derived from TCGA and GTEx datasets. In details, we analyzed the differential expression of SCARB1, MAPK3K1, and S100A10 between tumor and normal tissues by using the "PRAD" dataset. We used UALCAN software and selected the prostate cancer dataset from TCGA to analyze the correlation between Gleason scores and the three selected gene expressions as well as other clinico-pathological characteristics. The P value cutoff value was 0.05. Student's t-test was performed to calculate a P value for expression analysis. The "Survival Analysis" module supplied a Kaplan-Meier curve that represents the relationship between the three genes and survival prognosis.
